# Glial-mediated immune modulation in glaucomatous neurodegeneration: mechanisms and therapeutic implications

**DOI:** 10.3389/fimmu.2025.1640110

**Published:** 2025-12-02

**Authors:** Fangwei Zong, Jiaxin You, Hong Wu, Xuerui Wang

**Affiliations:** 1 Department of Ophthalmology, The Second Hospital of Jilin University, Changchun, Jilin, China; 2 Department of Clinical Laboratory, The Second Hospital of Jilin University, Changchun, Jilin, China

**Keywords:** glaucoma, neuroinflammation, microglia, astrocytes, Müller cells, TNF-α, complement, TLR4

## Abstract

Glaucoma, a leading cause of irreversible blindness, is characterized by retinal ganglion cell (RGC) degeneration and optic nerve damage. While elevated intraocular pressure (IOP) is a major risk factor, emerging evidence highlights neuroinflammation as a critical driver of disease progression. Glial cells, particularly microglia, astrocytes, and Müller cells, are central to this inflammatory process, orchestrating immune responses through the release of cytokines, chemokines, and complement proteins. Microglia and astrocytes contribute to early inflammatory amplification through tumor necrosis factor-alpha (TNF-α), complement, and Toll-like receptor 4 (TLR4) pathways, while Müller cells further promote tissue damage via ATP/P2X7R signaling and senescence-associated mechanisms. Leukocyte infiltration, triggered by glial-derived chemokines and matrix metalloproteinases (MMPs), underscores the intersection of innate and adaptive immunity in glaucoma. Importantly, preclinical studies demonstrate that targeting neuroinflammatory pathways confers RGC protection, thus modulating glial activation and immune signaling represents a promising therapeutic strategy for glaucoma, particularly in IOP-refractory cases. This review synthesizes current knowledge on the role of glial cells in initiating and perpetuating immune responses that exacerbate RGC loss, and details how activated microglia and astrocytes release pro-inflammatory mediators and upregulate pathogenic signaling pathways.

## 1 Introduction

Glaucoma, a progressive optic neuropathy, involves retinal ganglion cell (RGC) apoptosis and axonal degeneration (1). Its pathogenesis is multifactorial, driven by elevated intraocular pressure (IOP), aging, oxidative stress, and genetic predisposition, yet mounting evidence identifies neuroinflammation and immune dysregulation as pivotal contributors to disease progression (2, 3). The lamina cribrosa, the principal site of injury, exhibits marked glial activation and inflammatory remodeling in both human and experimental models, where inhibition of glial activation and cytokine signaling preserves RGC integrity and optic nerve structure (4, 5). Clinically, optic disc hemorrhages and peripapillary chorioretinal atrophy may reflect secondary manifestations of glia-driven neuroinflammation (6, 7).

Studies have shown that activation of resident glial cells in the retina, including microglia, astrocytes, and Müller cells, and infiltration of peripheral immune cells such as T lymphocytes, B lymphocytes, and regulatory T cells (Tregs), play pathogenic roles and are closely associated with RGC loss (8–10). Activated microglia release tumor necrosis factor-alpha (TNF-α), interleukin (IL)-1β, and complement components that propagate inflammatory cascades and sensitize RGCs to injury (8, 9). Astrocytes amplify these immune responses through Toll-like receptor (TLR) activation, NF-κB signaling, and chemokine secretion, whereas Müller cells contribute to retinal immune modulation by releasing ATP, IL-6, and matrix metalloproteinases (MMPs), which facilitate leukocyte infiltration and tissue remodeling. This coordinated glia—immune axis establishes a self-perpetuating inflammatory loop that drives chronic neurodegeneration (8, 11). Notably, pharmacological modulation of these pathways demonstrates therapeutic promise. For example, rapamycin exerts neuroprotective effects not solely through inhibition of microglia activation but also by suppressing mTOR-dependent immune activation and cytokine release, underscoring the immunoregulatory dimension of glial targeting (12–14). The convergence of glial activation and immune signaling thus represents a central mechanism linking ocular hypertension to neurodegenerative pathology.

In summary, this review synthesizes current advances on how glial cells—microglia, astrocytes, and Müller cells—mediate immune modulation in glaucomatous neurodegeneration. It further highlights the molecular pathways by which glial-derived cytokines, chemokines, and complement proteins orchestrate retinal immune responses, contributing to RGC loss and optic nerve damage.

## 2 Central nervous system immune cells and glaucoma

Glial cells in the retina and optic nerve head (ONH) are broadly classified into microglia (the resident immune cells of the central nervous system (CNS) and macroglia, which include astrocytes and Müller cells (15, 16). Microglia continuously survey the microenvironment, respond rapidly to injury, and orchestrate immune responses. In contrast, astrocytes and Müller cells maintain structural integrity, regulate extracellular ion homeostasis, and provide metabolic support to neurons (17). Under glaucomatous stress, these glial cells undergo activation and phenotypic shifts that transform them into neurotoxic effectors, contributing to progressive RGC loss (11).

### 2.1 Microglia

Microglia are resident immune cells within the retina that enter through the pars plana of the ciliary body and the optic nerve head (18, 19). Under physiological conditions, these cells play a crucial role in preserving retinal equilibrium by clearing cellular waste through phagocytic mechanisms (20, 21). Progressive loss of RGCs is a pathological hallmark of glaucoma, with microglia actively participating in RGC damage and immune-inflammatory responses (22). Importantly, alterations in microglial morphology and gene expression profiles emerge prior to observable RGC degeneration and measurable declines in visual function loss in glaucoma (23). As the disease advances, microglia transition into an activated state, adopting a neurodegenerative phenotype that promotes neuronal toxicity, ultimately exacerbating RGC injury and apoptosis (24, 25). Since the optic nerve constitutes a critical component of the CNS, research by Liu et al. (26) demonstrated that infrared stimulation of the CNS results in microglial activation, enhanced phagocytic activity, and release of multiple pro-inflammatory factors, including IL-1α, IL-1β, IL-6, reactive oxygen species (ROS), and TNF-α (27). Such modifications foster a pro-inflammatory CNS microenvironment that hastens RGC depletion. Retinal gap junctions (GJs) serve as vital neuroprotective structures for RGCs. In a microbead-induced ocular hypertension (OHT) murine glaucoma model, Kumar et al. (28) observed that GJ inhibition occurs concurrently with microglial activation and RGC degeneration, implying a causative relationship between microglial reactivity and RGC loss.

### 2.2 Astrocytes

Astrocytes, the predominant non-neuronal glial population within the retinal nerve fiber layer, ganglion cell layer, and optic nerve, are indispensable for maintaining retinal homeostasis by restraining RGC and axonal degeneration (29). Astrocytic injury has been shown to induce degeneration of RGCs and their axons, contributing to glaucomatous vision loss (30). Additionally, deformation and remodeling of the lamina cribrosa (LC), the principal structural component of the optic nerve head, can damage both the traversing optic nerve fibers and capillaries, thus acting as a critical pathological factor in glaucoma progression (31). Disruption of astrocytic integrity precipitates RGC loss and axonal damage, thereby driving glaucomatous vision decline (32, 33), indicating that astrocytic dysfunction has direct consequences for optic nerve stability and may initiate or exacerbate glaucomatous pathology. Under pathological stress, astrocytes can undergo a phenotypic shift toward a neurotoxic, pro-inflammatory state (34). This phenotypic transition is driven by microglia-derived mediators, notably interleukin (IL)-1α, tumor necrosis factor (TNF)-α, and complement component C1q (35). Given the central role of activated microglia in glaucoma pathogenesis, microglia-driven astrocytic polarization is likely a key driver of RGC apoptosis and optic nerve injury (36). Joshi et al. (37) demonstrated that mitochondrial fragments released by microglia or *in vitro* preconditioning with IL-18 induces a neurotoxic astrocytic phenotype detrimental to RGC viability.

### 2.3 Müller cells

Müller cells, the principal macroglial cells of the retina extending across its entire thickness, are indispensable for maintaining retinal homeostasis but exhibit profound dysfunction in glaucomatous pathology, with their presence and activation confirmed in human and experimental glaucoma models (38, 39). A hallmark of their reactive state is the upregulation of glial fibrillary acidic protein (GFAP), prominently observed in the glaucomatous optic nerve head (40). Pathological accumulation of extracellular ATP, a common feature in glaucoma, activates Müller cells via purinergic P2 receptors (P2Rs), initiating a feed-forward loop of additional ATP release. Given that RGCs express the high-threshold purinergic receptor P2X7R (32, 41), Müller cell–derived ATP can bind to RGC P2X7R, eliciting sustained calcium influx that perturbs intracellular calcium homeostasis (42, 43). This calcium overload promotes the opening of the mitochondrial permeability transition pore (mPTP), leading to mitochondrial depolarization, cytochrome c release, and the activation of calcium-dependent proteases such as calpains, ultimately culminating in caspase activation and apoptotic RGC death (44, 45). Müller-derived ATP promotes RGC injury via P2X7R-mediated calcium overload and apoptosis (41, 46). Müller–microglia crosstalk further amplifies retinal inflammation. In chronic ocular hypertension models, reactive Müller cells activate microglia through ATP/P2X7R signaling, stimulating the production of pro-inflammatory cytokines such as TNF-α and IL-6 (8). These cytokines, in turn, act on Müller cells to intensify inflammatory responses, with NF-κB signaling serving as a central mediator (47). The anti-inflammatory SIX1 gene is downregulated in glaucomatous Müller cells (48), suggesting its loss potentiates inflammatory cascades. Age-related mechanisms further link Müller cells to glaucoma. Epidemiological data associate glaucoma prevalence with aging (49), while the glaucoma-risk gene SIX6, overexpressed in glaucomatous Müller cells and astrocytes, drives senescence via p16INK4 upregulation (50, 51). These converging inflammatory, neurotoxic, and senescence-associated pathways underscore Müller cells as central effectors in glaucoma pathogenesis, irrespective of whether the initiating insult arises from RGCs, the optic nerve, or systemic aging processes (52, 53). Notably, glial cells operate not in isolation but through dynamic crosstalk. These interactions exemplify how glial communication drives a feed-forward neuroinflammatory circuit that accelerates RGC injury and optic nerve damage (54, 55). Recognizing this network-level integration is key to identifying therapeutic strategies that target glial synergy rather than isolated cell types.

## 3 Mechanistic role of glial cells in glaucoma pathogenesis

### 3.1 Activation of astrocytes, microglia, and Müller cells

Astrocytes and microglia, the principal glial cell populations within the retina and ONH, undergo rapid activation during the earliest stages of glaucomatous pathology (56, 57). Astrocytes, concentrated within the retinal nerve fiber layer and ganglion cell layer, are especially abundant at the lamina cribrosa, where they provide essential structural and metabolic support for RCGs (58). However, it remains debated whether glial activation serves as an initiating insult or a downstream effector in glaucoma pathogenesis. Evidence suggests that glial reactivity is not sufficient by itself to induce glaucomatous neurodegeneration, as substantial glial activation is also observed in certain ocular inflammatory models without subsequent RGC loss or optic nerve damage. Instead, elevated IOP and aging—both established glaucoma risk factors—appear to be critical upstream modulators that sensitize glial cells toward a pathogenic phenotype (11, 59). Notably, prior to detectable RGC axon damage, the expression of genes and proteins related to astrocytic activation, including pattern recognition receptor (PRR)–associated adaptor proteins and effector molecules, is markedly upregulated (60, 61). This includes enhanced complement deposition, epidermal growth factor receptor (EGFR) expression, and increased levels of pro-inflammatory mediators such as inducible nitric oxide synthase (iNOS) and cyclooxygenase-2 (COX-2) (62). Activated microglia similarly exhibit heightened expression of inflammatory cytokines, complement components, and major histocompatibility complex (MHC) molecules (63), while facilitating the recruitment of circulating immune cells into the ONH, thereby amplifying neuroinflammatory cascades (64). Müller cells, which span the entire retinal thickness, are also activated in response to intraocular pressure elevation and neurodegenerative stress, contributing to the early pro-inflammatory milieu through ATP release and P2X7R signaling cascades. Their activation promotes microglial reactivity and amplifies retinal inflammation via NF-κB–dependent pathways (8). Neuroinflammation driven by glial cells activation in the glaucomatous ONH may lower neuronal stress thresholds and promote neuronal injury (65, 66), culminating in glial scar formation and inhibition of RGC axonal regeneration (50). Although transient or moderate glial activation can confer neuroprotection, through trophic factor release and metabolic support, prolonged or excessive activation transitions these cells into chronic inflammatory phenotypes, thereby accelerating progressive neurodegeneration (67).

### 3.2 Inflammatory signaling pathways activated by glial cells

#### 3.2.1 TLR-mediated neuroinflammatory signaling

Toll-like receptors (TLRs), a pivotal subset of pattern recognition receptors (PRRs), serve as sentinels of the innate immune system by recognizing pathogen-associated molecular patterns (PAMPs) and damage-associated molecular patterns (DAMPs) (54, 68). Distinct TLR family members display ligand specificity; for example, TLR3 is activated by viral double-stranded RNA, whereas TLR4 senses endogenous stress signals such as heat shock proteins (HSPs) (69, 70). In the central nervous system, microglia express the full complement of TLRs, while astrocytes selectively express TLR2, TLR3, TLR4, TLR5, and TLR9, each attuned to discrete PAMP or DAMP signatures (71, 72). In glaucomatous retina, TLR expression is markedly elevated (73). *In vitro* studies reveal that both HSPs and oxidative stress act as potent inducers of TLR expression, thereby amplifying the release of pro-inflammatory cytokines and immunostimulatory mediators that activate both innate and adaptive immune pathways (74, 75). Notably, tenascin-C, an endogenous ligand for TLR4, is upregulated in glaucomatous ONH and has been shown to activate TLR4 signaling in arthritis (76, 77). It is hypothesized that tenascin-C may initiate inflammation via TLR4 prior to DAMP release by injured RGCs (78, 79). TLR activation converges on two principal signaling axes. The myeloid differentiation primary response 88 (MyD88)-dependent pathway activates nuclear factor-κB (NF-κB) and activator protein-1 (AP-1), driving transcriptional upregulation of TNF-α, IL-1, IL-6, and a spectrum of chemokines (80, 81). Alternatively, the Toll/IL-1 receptor (TIR)-domain-containing adaptor-inducing interferon-β (TRIF)-dependent pathway predominantly engages interferon regulatory factors (IRFs) (82). Caffeic acid phenethyl ester suppresses glial activation and migration, inhibits NF-κB-mediated inflammation, and protects RGCs from degeneration (12).

#### 3.2.2 TNF-α mediated neuroinflammatory signaling

**Figure 1:**
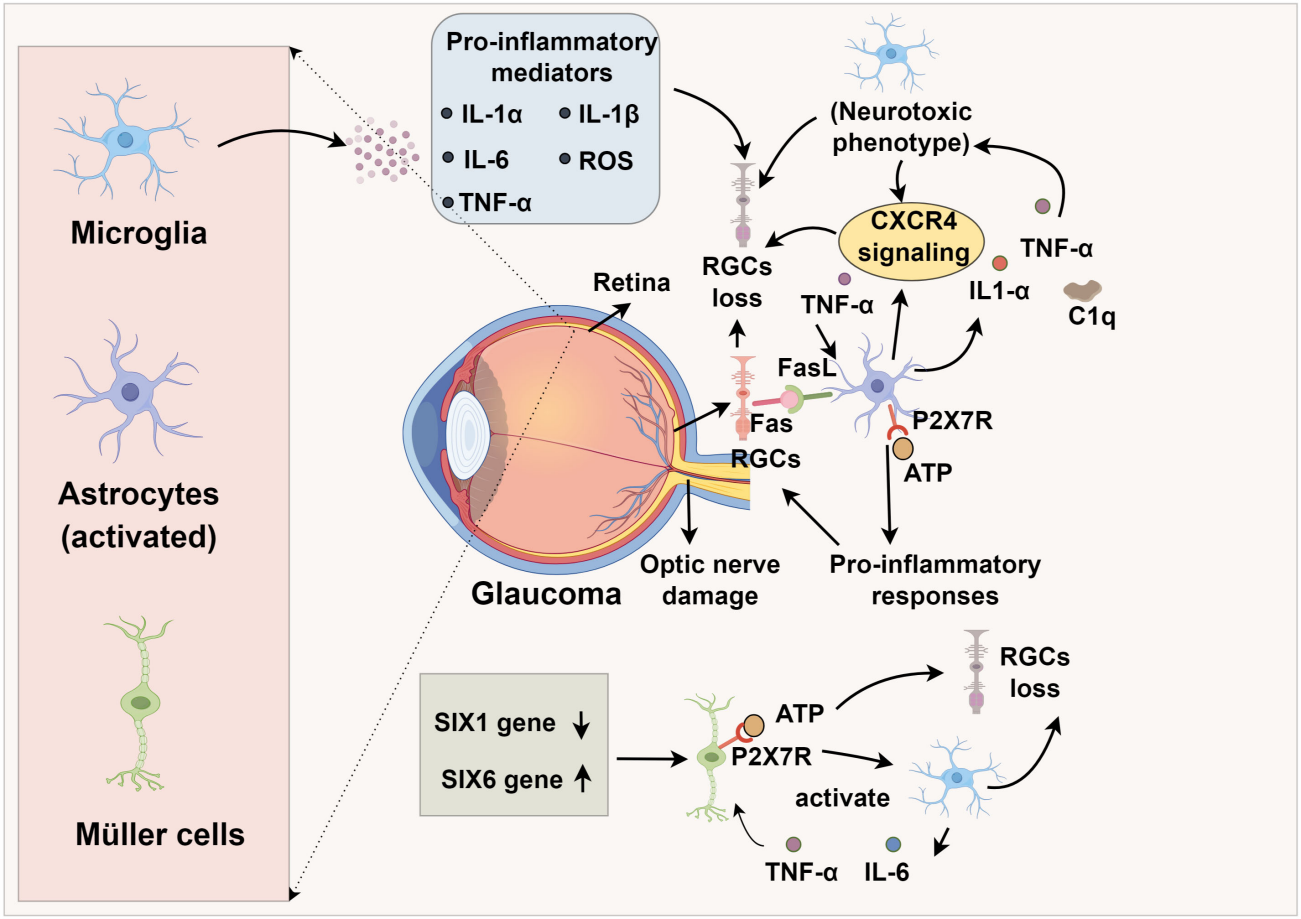
Glail activation and immune crosstalk in glaucomatous optic nerve degeneration.

TNF-α, a central pro-inflammatory cytokine in neurodegeneration, is secreted by both astrocytes and microglia, with astrocytes constituting the predominant source in the ONH (83, 84). In glaucomatous retina and ONH, TNF-α and its primary receptor TNF receptor 1 (TNF-R1) are markedly upregulated, with elevated expression detected in RGCs and their axons (85). Despite some evidence suggests that TNF-α may exert transient neuroprotective effects during the initial stages of optic nerve injury, the preponderance of experimental data implicates it in promoting RGC death through TNF-R–dependent caspase activation, mitochondrial dysfunction, and oxidative stress (86). Binding of TNF-α to TNF-R1 activates the TNF receptor-associated death domain (TRADD), the TNF receptor-associated factor (TRAF) superfamily, and various kinases, culminating in caspase-mediated apoptosis in RGCs (87, 88). Additionally, soluble TNF-α may promote CP-AMPAR (Ca^2+^-permeable AMPA receptor) expression in RGCs, exacerbating excitotoxicity (89, 90). TNF-α can also induce RGC death via the FasL pathway, as intraocular injection of TNF-α leads to RGC loss, which is attenuated by FasL inhibition (91). Pharmacological or genetic TNF-α inhibition mitigates microglial activation, axonal degeneration, and RGC loss (92, 93). In corneal chemical injury models, TNF-α blockade reduces monocyte infiltration into the retina and microglial activation, thereby decreasing the incidence of secondary glaucoma and RGC death (94). TNF-α also activates c-Jun N-terminal kinase (JNK), NF-κB, and extracellular signal-regulated kinase (ERK) pathways, further potentiating glia-mediated inflammation via IL-1 upregulation (95, 96) (**Figure 1**).

#### 3.2.3 Complement activation

Complement activation constitutes another key early inflammatory event (97). Elevated complement levels are detected in glaucomatous retinas, particularly at the ONH and inner retinal layers (98, 99). Activation of retinal astrocytes correlates with increased C1q expression in RGCs (100). In a genetic model of glaucoma, Shinozaki et al. (30) reported that elevated IOP was accompanied by retinal C1q upregulation and RGC apoptosis. RGCs can sense damage and activate C1, triggering a cascade that activates C3 and C5 and recruits immune cells to the injury site (101, 102). Importantly, the role of C3 in glaucoma appears to be context dependent, showing a duality between early neuroprotection and late-stage neurotoxicity (103). Complement factors have dual roles in glaucoma (104). C1qa is expressed in ONH microglia and RGC dendrites; its inhibition reduces dendritic and synaptic loss in glaucoma (105). Moreover, membrane attack complexes (MACs) accumulate in the ONH and RGCs, and MAC inhibition reduces RGC apoptosis (104). Recent evidence suggests that in the early phase of disease, astrocyte-derived C3 can signal through the epidermal growth factor receptor (EGFR) pathway, promoting astrocytic survival responses and metabolic support to stressed axons (106). This transient EGFR–C3 interaction may act as a compensatory mechanism, particularly under acute intraocular pressure (IOP) elevation, to preserve tissue integrity (107, 108). However, with sustained or chronic activation, persistent C3 cleavage leads to excessive production of downstream complement fragments (C3b, C5b-9) and ultimately the formation of MAC (109, 110). MAC deposition on RGC somas and axons contributes to neurodegeneration by inducing membrane pore formation and triggering inflammatory cell recruitment (111). Although Harder et al. (106) suggest that early astrocytic C3/EGFR signaling can be beneficial, other studies indicate that in chronic glaucoma, prolonged complement activity becomes detrimental. This switch depends on the timing of activation, the cellular source (astrocyte-derived C3 and microglia-driven complement cascade), and the local inflammatory milieu, which collectively determine whether complement exerts neuroprotective or neurotoxic effects (112–114). Ischemia-reperfusion increases retinal complement expression and deposition, while C3 knockout reduces optic nerve damage and increases RGC survival (115, 116). Bosco et al. (117) demonstrated that intravitreal AAV2.CR2-Crry injection reduces retinal C3d deposition and protects RGC axons and soma under chronic ocular hypertension.

#### 3.2.4 EGFR/iNOS/COX-2 pathways

EGFR expression and tyrosine phosphorylation are elevated in activated ONH astrocytes, promoting the production of iNOS, COX-2, and prostaglandins (PGs), thereby affecting RGC survival and ONH structure (118, 119). In high IOP mouse models, both neuronal nitric oxide synthase (nNOS) and iNOS are elevated in astrocytes at the ONH and retinal layers (120). Neufeld et al. (121) linked axonal damage to excessive nitric oxide (NO) production. While eNOS and nNOS are constitutively expressed in normal ONH astrocytes and vasculature, iNOS is upregulated by day 4 of elevated IOP and persists for months. Aminoguanidine, an iNOS inhibitor, significantly reduces RGC loss and may offer neuroprotection in glaucoma (122). Zhang et al. (119, 123) showed that while COX-2 is undetectable in normal ONH, it becomes detectable within 24 hours in cultured explants and peaks at 3 days. EGF induces COX-2 expression and PGE2 synthesis in astrocytes in a time-dependent manner, which is blocked by EGFR inhibitor AG1478. Downstream, ERK and p38 pathways also regulate COX-2/PGE2 production via EGFR signaling. COX-2 oxidizes arachidonic acid to produce PGs; PGD2, PGE2, and PGI2 may exert neuroprotective effects via DP1, EP2/EP4, and IP receptors, whereas PGE2 and PGF2α can induce neurotoxicity via EP1 and FP receptors (123, 124). Although substantial evidence implicates the EGFR/iNOS/COX-2 axis in glaucomatous neuroinflammation, the temporal dynamics and context-dependent roles of individual inflammatory mediators across disease stages remain incompletely understood, warranting further investigation.

#### 3.2.5 JAK/STAT-mediated inflammatory signaling

The Janus kinase/signal transducer and activator of transcription (JAK/STAT) pathway is a key regulator of glial-mediated neuroinflammation and has been increasingly implicated in glaucomatous neurodegeneration (125). Upon cytokine binding—particularly IL-6, IL-10, and interferons—glial cell–expressed receptors activate JAK kinases, leading to phosphorylation and nuclear translocation of STAT proteins, which then drive transcription of pro- or anti-inflammatory genes depending on the cellular context (126). In glaucomatous retina, STAT3 is the most prominently activated STAT protein in astrocytes and Müller cells and is responsible for upregulating genes involved in gliosis (GFAP), cellular stress responses, and cytokine amplification (IL-6, SOCS3). Notably, elevated STAT3 phosphorylation has been documented in the optic nerve head of both rodent models and human glaucoma tissues, suggesting a conserved role in glial activation and RGC injury (127, 128). Inhibition of JAK2 or STAT3 pharmacologically (with AG490 or Stattic) mitigates gliosis, preserves RGC function, and reduces optic nerve damage in experimental models of chronic ocular hypertension (127). However, the JAK/STAT pathway exhibits dual roles: transient STAT3 activation may promote glial neuroprotective programs, while sustained or excessive activation promotes gliosis and neurotoxicity (11, 126). Importantly, crosstalk with NF-κB and PI3K/AKT pathways further integrates STAT3 into a complex signaling hub that fine-tunes glial responses under stress conditions (129). Thus, therapeutic targeting of the JAK/STAT pathway requires precise temporal and cell-specific modulation to avoid disrupting beneficial glial responses.

### 3.3 Leukocyte infiltration and RGC death triggered by glial activation

#### 3.3.1 Immune cells infiltration in glaucoma

Astrocyte-derived matrix metalloproteinases (MMPs) may degrade the basement membrane and compromise the glial lamina at the glaucomatous ONH (130). Leukocyte transendothelial migration is among the earliest detectable changes in DBA/2J glaucoma mouse models (131). In healthy optic nerves, CD163^+^ macrophages are sparsely distributed along axonal septa, whereas both early- and late-stage glaucoma show increased infiltration of CD163^+^ macrophages into the nerve bundles (132). The accumulation and activation of macrophages and microglia within the optic nerve have been documented across multiple glaucoma models and are considered pivotal contributors to early disease pathogenesis (103, 133, 134). Transcriptomic profiling by Howell and Johnson revealed early-stage upregulation of selectins, adhesion molecules, and chemokines in glaucomatous ONH, which collectively facilitate leukocyte recruitment prior to overt axonal injury. Experimental depletion of monocytes via targeted irradiation prevents optic nerve damage, whereas restoration of injury following endothelin-2 (ET-2) administration underscores the causal role of immune cell infiltration in glaucomatous neurodegeneration (135). Glycosylation-dependent cell adhesion molecule 1 (GlyCAM-1) may facilitate monocyte trafficking to the ONH (136). In addition to macrophages, T lymphocytes have emerged as critical contributors to glaucomatous immune pathology (137). Glial cells upregulate MHC class II molecules in glaucomatous human and experimental retinas, enabling antigen presentation and promoting T-cell activation (11, 137, 138). Glia-derived chemokines such as CCL2 and CXCL10 recruit T cells to the ONH, where infiltrating T cells release IFN-γ, TNF-α, and other pro-inflammatory mediators that further activate resident glial populations. This reciprocal amplification loop—where glial cytokines attract T cells, and T cells in turn enhance glial reactivity—forms a self-sustaining inflammatory circuit that exacerbates RGC injury (120, 137). Within the CNS, TNF-α activates endothelial cells, promoting integrin-dependent leukocyte migration (139). Astrocytes, which express multiple integrin isoforms, exhibit elevated perivascular integrin expression in response to elevated IOP. This upregulation facilitates extracellular matrix (ECM)–cytoskeleton coupling and promotes cellular migration, adhesion, differentiation, and pro-inflammatory signaling (140, 141).

#### 3.3.2 RGC death triggered by glial activation

The membrane-bound form of Fas ligand (FasL) has been identified as a key effector in RGC apoptosis in glaucomatous mouse models (91). Krishnan et al. (21) found that activated microglia release TNF-α, which upregulates FasL expression on microglia in glaucomatous retinas, enhancing FasL-Fas binding to RGCs and thereby directly triggering apoptosis and exacerbating glaucoma progression. Moreover, microglial activation is associated with upregulation of the apolipoprotein E (ApoE) gene and the galectin-3 (Lgals3) gene (142). Knockout of ApoE in glaucomatous mouse models prevented RGC loss and suppressed the expression of neurodegenerative genes including Lgals3, indicating that ApoE-related microglial activation contributes to disease progression (142). The ApoE–Lgals3 signaling axis thus represents a potential therapeutic target for glaucoma. Importantly, ApoE also serves as an endogenous ligand for the microglial receptor TREM2, activating the DAP12–SYK signaling cascade that drives metabolic reprogramming, proliferation, and a disease-associated microglial (DAM) phenotype (143–145). In this context, upregulated Lgals3 reinforces the TREM2-ApoE axis, amplifying pro-inflammatory gene expression and promoting phagocytic activity that, while initially protective, becomes maladaptive and neurotoxic (146). Concurrently, these changes are associated with the downregulation of the homeostatic CX3CR1–CX3CL1 axis, which ordinarily restrains microglial overactivation (147, 148). The combined effect is a shift toward chronic microglial activation and synaptic toxicity, accelerating RGC degeneration. These intersecting pathways highlight a mechanistic bridge between ApoE-Lgals3 and canonical TREM2/CX3CR1 signaling in shaping microglial responses during glaucomatous neurodegeneration (149).

Extracellular ATP serves as a potent astrocytic activator (150, 151). At high concentrations, ATP activates purinergic receptor P2X7R, a high-threshold subtype of the P2 receptor (P2R) family, which induces pro-inflammatory responses in astrocytes (152, 153). P2X7R activation triggers the release of chemokines, cytokines, and ROS, thereby amplifying RGC injury, and also stimulates cytokine production in Müller cells (154, 155). Furthermore, chemokine signaling pathways contribute to astrocyte-mediated neurotoxicity. Enhanced CXCR4 activation in astrocytes or microglia facilitates glutamate release from astrocytes, provoking excitotoxic injury and promoting RGC degeneration and necrosis (30). In normal-tension glaucoma (NTG) mouse models, retinal astrocytes exhibit upregulation of CXCL-12, the endogenous agonist of CXCR4 (156). Collectively, these findings underscore astrocytes as both immune effectors and direct mediators of neuronal injury, positioning astrocytic activation and phenotypic modulation as central mechanisms in glaucoma pathogenesis (**Table 1**).

**Table 1:** Glial cell–mediated immune mechanisms and their roles in glaucomatous neurodegeneration.

Glial cell type	Activation stimuli	Key immune mediators released	Signaling pathways	Downstream effects on retina/ONH	Functional consequences
Microglia	Elevated IOP, oxidative stress, RGC-derived DAMPs	TNF-α, IL-1β, IL-6, C1q, C3, ROS	TLR4–MyD88/NF-κB, TNF-α–FasL, CX3CR1–CX3CL1, ApoE–TREM2/Lgals3	Cytokine amplification, complement activation, MHC II upregulation, leukocyte recruitment	Early RGC apoptosis, immune cell infiltration, synaptic stripping
Astrocytes	Microglial cytokines (TNF-α, IL-1α), mitochondrial fragments, tenascin-C	IL-6, CXCL10, TNF-α, iNOS, COX-2, C3	TLR4–NF-κB, EGFR–iNOS–COX-2, STAT3, JNK/ERK	Upregulation of adhesion molecules, basement membrane degradation (via MMPs), antigen presentation (MHC II)	Glial scar formation, RGC excitotoxicity, leukocyte transmigration
Müller Cells	ATP, hypoxia, aging, inflammatory cytokines	ATP, IL-6, MMPs, ROS	P2X7R–Ca^2+^ overload–mPTP, NF-κB, STAT3, SIX6–p16INK4 pathway	Induction of microglial reactivity, glia–glia inflammatory loops, senescence-associated inflammation	RGC mitochondrial dysfunction, apoptosis, age-linked degeneration
Cross-talk	Glial-glial and glial-immune cell interaction	TNF-α, IL-6, CCL2, CXCL12, FasL	TNF-α–TNF-R1, CXCL12–CXCR4, Fas–FasL	Positive feedback loop in inflammation, glia-mediated T cell activation	Sustained neuroinflammation, loss of homeostatic restraint, chronic neurodegeneration

IOP, intraocular pressure; RGC, retinal ganglion cell; ONH, optic nerve head; DAMPs, damage-associated molecular patterns; ROS, reactive oxygen species; TLR, Toll-like receptor; NF-κB, nuclear factor kappa-light-chain-enhancer of activated B cells; MHC, major histocompatibility complex; EGFR, epidermal growth factor receptor; iNOS, inducible nitric oxide synthase; COX-2, cyclooxygenase-2; MMPs, matrix metalloproteinases; mPTP, mitochondrial permeability transition pore.

## 4 Conclusion

Glial activation and immune modulation are central drivers of glaucomatous neurodegeneration, operating through tightly interlinked inflammatory networks within the optic nerve head. Activated microglia, astrocytes, and Müller cells release cytokines, chemokines, complement components, and ROS which lead to RGC injury and recruit peripheral immune cells, further amplifying tissue damage. These responses are further shaped by key signaling pathways, including TLR4–NF-κB, TNF-α–FasL, complement C3/C5–MAC formation, and EGFR/iNOS/COX-2 cascades, as well as age-related senescence programs. Chronic or excessive activation transforms initially protective glial responses into maladaptive, neurotoxic states, driving progressive RGC apoptosis, lamina cribrosa remodeling, and irreversible vision loss.

Future therapeutic strategies should prioritize temporally targeted modulation of glial activation, inhibition of pathogenic inflammatory pathways, and disruption of maladaptive glia–immune interactions. Integrating longitudinal biomarker profiling with advanced imaging could enable stage-specific interventions, particularly in early glaucoma when neuroprotection is most feasible. Moreover, the convergence of neuroinflammatory mechanisms in glaucoma with other central nervous system disorders suggests that repurposing or co-developing glia-targeted agents may accelerate translational progress. Such approaches hold the potential to expand treatment paradigms beyond intraocular pressure control, offering new avenues for preserving vision.
